# Isolation and Characterization of a Porcine Transmissible Gastroenteritis Coronavirus in Northeast China

**DOI:** 10.3389/fvets.2021.611721

**Published:** 2021-03-02

**Authors:** Dongwei Yuan, Zihan Yan, Mingyue Li, Yi Wang, Mingjun Su, Dongbo Sun

**Affiliations:** ^1^College of Animal Science and Veterinary Medicine, Heilongjiang Bayi Agricultural University, Daqing, China; ^2^Daqing Center of Inspection and Testing for Agricultural Products Ministry of Agriculture, Daqing, China

**Keywords:** transmissible gastroenteritis virus, virus isolate, phylogenetic analysis, pathogenicity, coronavirus

## Abstract

Transmissible gastroenteritis virus (TGEV) is a coronavirus (CoV) that is a major pathogenity of viral enteritis and diarrhea in suckling piglets, causing high morbidity and mortality. In this study, a TGEV strain HQ2016 was isolated from northeast China and characterized its genome sequence and pathogenicity. The phylogenetic analysis indicated that the TGEV HQ2016 strain was more similar to the TGEV Purdue cluster than to the Miller cluster. Both recombination and phylogenetic analysis based on each structural and non-structural gene revealed no recombination event in the HQ2016 strain. Experimental infection study using colostrum-deprived newborn piglets successfully showed that the HQ2016 can cause clinical symptoms including anorexia and yellow-to-whitish watery diarrhea, which are characteristics of TGE, in the inoculated piglets 48 h post-inoculation. These results provide valuable information about the evolution of the porcine CoVs.

## Introduction

Coronaviruses (CoVs) are the main etiological agents underlying outbreaks of porcine diarrhea, causing substantial economic losses (1). Transmissible gastroenteritis virus (TGEV) is a member of the family *Coronaviridae* that was first reported in 1946 in the USA (2). Since then, the disease always happened in swine-producing areas of the world (1, 3), and reported many times in China in recent years (4–8). Epidemiological investigations have shown that TGEV is often present in the spring and autumn in the northeast of China, sometimes in mixed infections with other diarrhea virus, and caused viral enteritis and severe diarrhea in all ages of pigs, especially with high mortality in suckling piglets (9, 10).

Transmissible gastroenteritis virus is an enveloped virus with a single-stranded, positive-stranded RNA genome of ~28.5-kb. The genome contains nine open reading frames (ORFs), which encode four structural proteins and five non-structural proteins: the spike glycoprotein (S); envelope protein (E); membrane glycoprotein (M); nucleocapsid protein (N); replicases 1a and 1b; ORF 3a and 3b proteins; and ORF 7 protein. The genes of TGEV are arranged in the order of 5′-rep-S-3a-3b-E-M-N-ORF7-3′ (4– 6). The mutation in the spikes protein may be an important indicator for evaluating the tropism and virulence of TGEV. The M protein is the main viral particle membrane protein, which is mainly embedded in the lipid vesicle membrane and is connected to the capsule during assembly of the virus nucleocapsid. The E protein is a transmembrane protein, and the N protein is exists in the viral membrane. The ORF3 is composed of two open frames ORF3a and ORF3b. ORF3a deletion is found in many TGEV strains and PRCV strain. The ORF7 counteracts host-cell defenses and affects the persistence of TGEV, and improves the survival rate of TGEV by negatively regulating the downstream caspase-dependent apoptotic pathways (5, 6, 11, 12).

In this study, we isolated a TGEV from clinical samples collected from farms in northeast China using PK15 cells, characterized its genome based on the whole-genome sequence, and investigated its pathogenicity in colostrum-deprived neonatal pigs in terms of a clinical assessment, viral shedding, virus distribution, histopathological changes, and a mortality analysis. The results suggested that we have isolated porcine enteric coronavirus TGEV HQ2016. The genetic characteristics and pathogenicity of this virus provided valuable information for the evolution of TGEV and will helpful research on the molecular pathogenesis of TGEV.

## Materials and Methods

### Specimen Collection and Screening

In 2016, a total of 50 intestine samples from piglets were collected from eight swine-raising farms in northeast China, in which the piglets showing watery diarrhea and dehydration and as known that all sow without any diarrhea viral vaccine inoculation. The intestinal samples were stored at −80°C. The samples were homogenized and diluted with sterile phosphate-buffered saline (PBS). The suspensions were repeatedly frozen and thawed three times, vortexed and clarified by centrifugation at 12,000 × g for 10 min at 4°C and the supernatants were filtered through 0.22 μm filters (Millipore, Billerica, MA, USA). Semi-nest reverse transcription (RT)-PCR (13) was used to identify the samples positive for TGEV, with two pairs of specific primers (TGEV-N-F: GGTAGTCGTGGTG- CTAATAATGA; TGEV-N-R1: CAGAATGCTAGACACAGATGGAA; TGEV-N-R2: GTT- CTCTTCCAGGTGTGTTTGTT).

### Virus Isolation and Plaque Purification

PK15 cells (American Type Culture Collection [ATCC] CCL-33) were cultured in Dulbecco's modified Eagle's medium (DMEM; Hyclone, USA) supplemented with 10% fetal bovine serum (FBS; Bovogen, Australia) at 37°C in a 5% CO_2_ incubator. Growth medium was removed from confluent monolayer cells; the cells were washed twice with DMEM and inoculated with a mixture of the supernatants of the positive tissue samples and DMEM containing 20 μg/ml trypsin (GIBCO, 1:250) at a ratio of 1:1. After adsorption for 60 min at 37°C, the cells were washed with DMEM, and maintenance medium consisting of DMEM supplemented with 10 μg/ml trypsin was added. The inoculated cell cultures were observed for CPE for 3–5 days, harvested, and blindly passaged for five times. The viruses in a CPE positive sample was cloned by repeating plaque purify three times and designated as HQ2016.

### Virus Titration With a Median Tissue Culture Infective Dose Assay

PK15 cells were seeded on 96-well plates and cultured overnight. The collected TGEV HQ2016 (passaged for 10 times) was 10-fold serially diluted, and used to inoculate cells, with eight replicates per dilution. The cells were then cultured continuously at 37°C under 5% CO_2_. The viral CPE was observed for 5–7 days. Tissue culture infective dose (TCID_50_) was determined with the Reed-Muench method (14) and expressed as TCID_50_ per milliliter.

### Indirect Immunofluorescence Assay

PK15 cells (1 × 10^6^) were seeded on six-well plates, cultured overnight, and then infected with TGEV HQ2016 (passaged for 10 times) at a multiplicity of infection (MOI) of 1.0. At 24 h after inoculation, the cells were fixed with 4% paraformaldehyde for 15 min and then per-meabilized with 0.2% Triton X-100 for 15 min. The cells were then blocked with 5% skim milk, and incubated overnight at 4°C with a TGEV-specific monoclonal antibody (5E8, supplied by Professor L. Feng, Harbin Veterinary Research Institute of the Chinese Academy of Agricultural Sciences, Harbin, China) diluted 1:1000. The cells were washed three times with PBS and incubated with a secondary antibody (fluorescein-isothiocyanate-conjugated goat anti-mouse IgG antibody, diluted 1:500) for 1 h at 37°C and then washed three times with PBS. The stained cells were visualized with fluorescence microscopy (Leica DMi8, Germany).

### Electron Microscopic Assay

Supernatants from plaque-purified TGEV HQ2016 (passaged for 8 times) infected cell cultures were concentrated by ultracentrifugation method. The supernatants of the cell cultures were centrifuged first at 6,000 × g for 30 min at 4°C, and then at 60,000 × g for 2 h at 4°C. After ultracentrifugation, the samples were negatively stained with 2% ammonium molybdate and adsorbed onto 300-mesh copper net for 2 min. The viral particles were examined with an electron microscope (Hitachi H7500, Tokyo, Japan).

### Extraction of Viral RNA and Complete Genome Sequencing

Culture supernatants from plaque-purified TGEV HQ2016 (passaged for 8 times) infected cells were collected and used for preparation of viral RNA. Total RNA was extracted using TRIzol Reagent (Invitrogen, Carlsbad, CA, USA), according to the manufacturer's instructions. The RNA samples were sent to testing company (Shanghai Probe Biotechnology Co., Ltd.) to determined complete genomic sequence with the Illumina high-throughput deep sequencing platform (15).

### Sequence Analysis

The sequences of TGEV reference strains used in this study were obtained from GenBank, as shown in Table 1. The nucleotide and the amino acid sequences of TGEV HQ2016 strain were compared with the corresponding sequences of the TGEV strains in the GenBank database. The sequence was analyzed using the computer program MEGA version 6.0 (16) and DNASTAR (17). Nucleotide and amino acid sequence identities were determined using the Clustal W program. To determine the relationships between representative TGEV isolates and HQ2016 strain, a phylogenetic tree based on the entire genome was constructed with the MEGA6.0 software through the neighbor-joining method. The reliability of the neighbor-joining tree was estimated by bootstrap analysis with 1,000 replicates.

**Table 1:** Information of the reference TGEV sequences used in this study in the database.

**No**.	**Isolate**	**Collected year**	**Country/Origin**	**GenBank accession no**.
1	SHXB	2013	China	KP202848.1
2	Purdue P115	2009	USA	DQ811788.1
3	PUR46-MAD	—	USA	AJ271965.2
4	WH-1	2011	China	HQ462571.1
5	AYU	2009	China	HM776941.1
6	Puedue	—	USA	NC_038861.1
7	HX	2012	China	KC962433.1
8	HE-1	2016	China	KX083668.31
9	SC-Y	2006	China	DQ443743.1
10	Z	2006	USA	KX900393.1
11	HB	1988	USA	KX900394.1
12	Mex-145	2018	USA	KX900402.1
13	Virulent Purdue	1952	USA	DQ811789.2
14	AHHF	2017	China	KX499468.1
15	TS	2016	China	DQ201447.1
16	JS2012	2012	China	KT696544.1
17	Miller M6	2009	USA	DQ811785.1
18	Attenuated H	2009	China	EU074218.2
19	H16	1973	China	FJ755618.2
20	HQ2016	2016	China	MT576083.1

### Recombination Analysis

We used the RDP4 software, including RDP, Bootscan, and SiScan, for a recombination analysis to detect the probable parental isolates and recombination breakpoints of TGEV HQ2016, with the default settings. The criteria used to detect recombination and identify breakpoints were *P* < 10^−6^ and a recombination score >0.6 (18).

### Pathogenicity of TGEV HQ2016 in Newborn Piglets

We used 12 newborn piglets of both sexes without colostrum, who had not been exposed to TGEV before and no anti-TGEV antibodies. The newborn piglets were randomly allocated to the control group (*n* = 6) or the challenged group (*n* = 6). The piglets were fed a mixture of skim milk powder (Inner Mongolia Yi Li Industrial Group Co., Ltd., China) and warm water. The groups were separated by room and ventilation system within the same facility. After acclimation for 1 day, the six piglets in the control group were orally administered 5 ml of DMEM and used as the uninfected controls. The six piglets in the challenged group were orally administered 5 ml of DMEM containing 5 × 10^6^ TCID_50_ of TGEV HQ2016 (passaged for 10 times). All the piglets were observed every 12 h for clinical signs of vomiting, diarrhea, lethargy, and altered temperature or body condition. Rectal swabs were collected from each piglet every 12 h and fecal consistency was scored. The grading standards for the clinical signs and fecal consistency are shown in Table 2. Fecal viral RNA shedding was detected with quantitative RT–PCR (19). The sequences of the primers used were: forward, 5′-AAACAACAGCAACGCTCTCG-3′; reverse, 5′-ATTGGCAACGAGGTCAGTGT-3′. The piglets in the two groups were sacrificed at 84 h after challenge. At necropsy, fresh samples of duodenum, jejunum, ileum, cecum, and colon were collected and fixed in 10% formalin solution. The fresh samples were stored at −80°C before a viral RNA distribution analysis with quantitative RT-PCR (19), and formalin-fixed samples were used for histopathological and immunohistochemical analyses. The mortality of the newborn piglets in each group was recorded daily.

**Table 2:** The grading standard for clinical symptom and feces of piglets.

**Scores**	**0**	**1**	**2**	**3**	**4**
Clinical symptoms	Normal	Slow movement, normal appetite	Lies, spirit languishes, loss of appetite	Difficult to walk, dehydration	Difficulty standing, dehydrated seriously and weight loss
Fecal consistency	Normal	Soft feces	Liquid with solid feces admixture	Watery feces	Watery diarrhea

### Statistical Analysis

The data, including the results of the clinical symptoms, fecal scores and viral load in which inoculated and control piglets, were compared among the different groups by one-way repeated measures ANOVA and the least significance difference (LSD). All data were processed and analyzed using SPSS21.0 Data Editor (SPSS Inc., Chicago, IL, USA). The results for the comparisons among groups were considered different if ^*^P < 0.05 or ^**^P < 0.01.

## Results

### Virus Isolation and Identification

**Figure 1:**
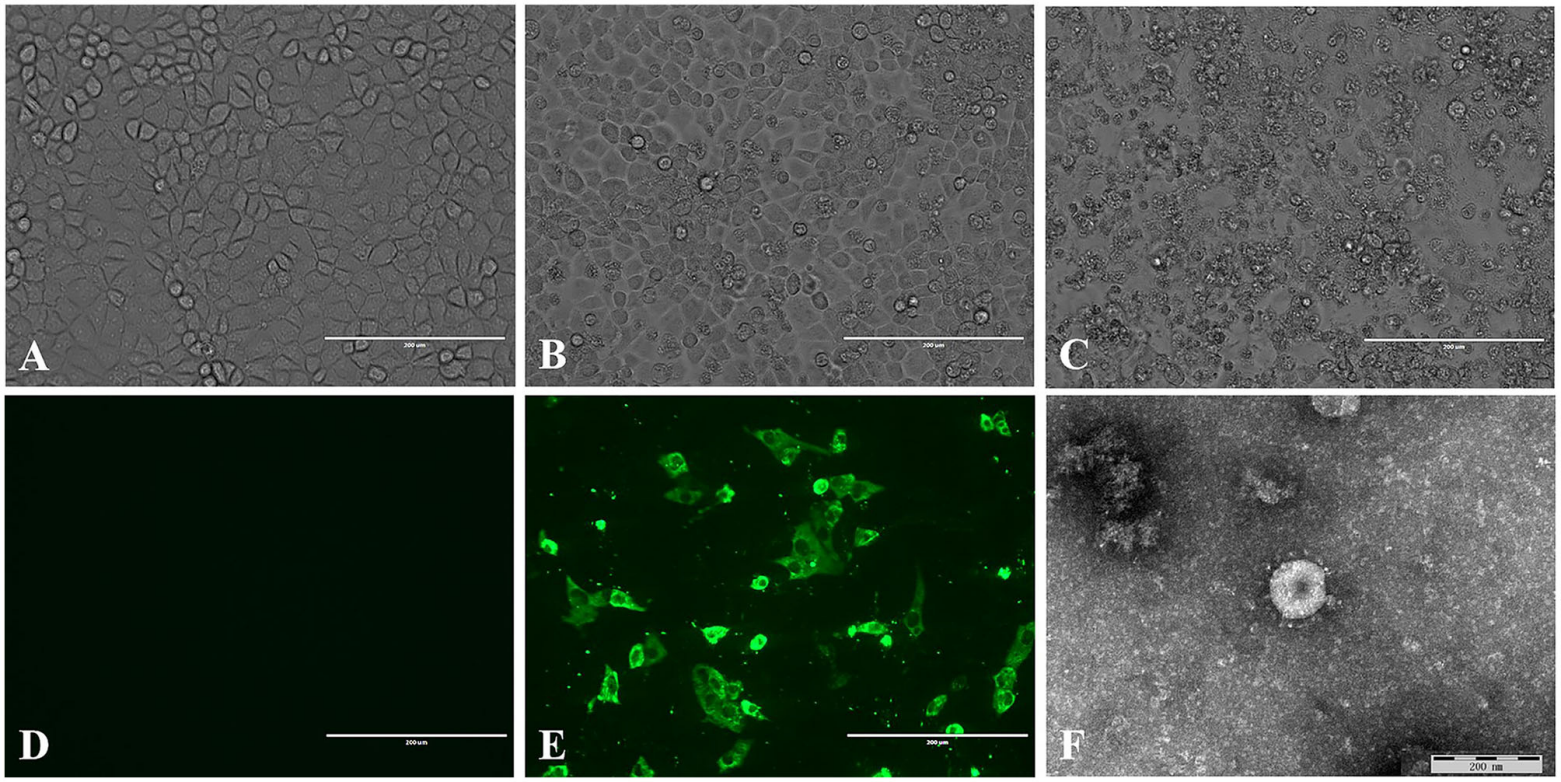
Isolation and identification of the TGEV HQ2016 strain. **(A)** Control (uninfected) PK-15 cells. **(B)** Cytopathic effect (CPE) induced by TGEV HQ2016 after infected 24 h in the PK-15 cell line. **(C)** Cytopathic effect (CPE) induced by TGEV HQ2016 after infected 36 h in the PK-15 cell line. **(D)** IFA identification of control (uninfected) PK15 cells. **(E)** IFA identification of TGEV HQ2016 infected PK15 cells. **(F)** Electron microscopy observation of TGEV HQ2016.

A total of 50 intestinal samples were collected from eight pig farms in northeast China. The piglets on these farms suffered vomiting and diarrhea. TGEV was detected in 20% of the samples, and the positive samples were from six farms. The supernatants of the TGEV-positive samples were used to inoculate PK15 cells, 6 of 10 positive samples were tested for virus isolation. Of which, three sample become positive CPE after five passages. No CPE was observed in control PK-15 cells (Figure 1A). The CPE was characterized by cell fusion, cell rounding and shrinkage, and the detachment of the cells into the medium (Figures 1B,C). TGEV antigen was identified in the cytoplasm of the virus inoculated PK-15 cells but not in mock inoculated cells by IFA using TGEV-specific monoclonal antibody (Figures 1D,E). Coronavirus-like particles with a diameter of 100 to 120 nm, similar to the size of TGEV were identified in the culture supernatant of the virus inoculated PK-15 cell by negative staining electron microscopy (Figure 1F). The virus isolate was designated as TGEV HQ2016 strain hereafter. And then, the titer of TGEV HQ2016 reached 10^5.25^ TCID_50_/0.1 ml at passage 10.

### Complete Genomic Sequence of TGEV Strain HQ2016

The genomic sequence of TGEV HQ2016 strain, determined with the illumina sequencing, platform was 28,571 nucleotides (nt) long, and the sequence was submitted to GenBank under accession number MT576083, and exhibited the genomic organization typical of all previously reported TGEV sequences, which are arranged in the order of 5′-rep-S-3a-3b-E-M-N-ORF7-3′ (4– 6). The 5′ portion of the genome contains a 303-nt untranslated region (UTR) which includes a potential short AUG-initiated ORF (nt 103–110), beginning with a Kozak sequence (5′-UCUAUGA-3′). The viral RNA-dependent RNA replicase include ORF1a (nt 304–12,357) and ORF1b (nt 12,315–20,357). Structural proteins encoding genes were S (nt 20,354–24,697), E (nt 25,846–26,094), M (nt 26,105–26,893), and N (nt 26,906–28,054), respectively. Non-structural protein encoding genes were ORF3a (nt 24,816–25,031), ORF3b (nt 25,125–25,859), and ORF7 (nt 28,029–28,265), respectively. The 3′end of the genome contains a 275-nt untranslated sequence and a poly(A) tail. The octameric sequence 5′-GGAAGAGC-3′ occurs upstream from the poly(A) tail.

### Genomic Characteristics

**Figure 2:**
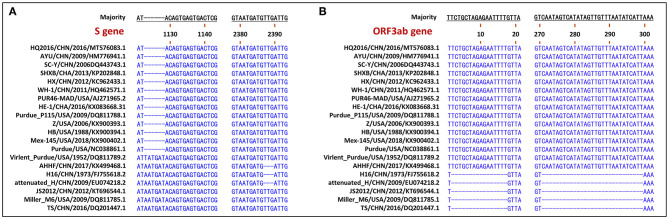
Visualization of genomic deletion regions in the 20 TGEV strains. **(A)** deletion regions of S gene. **(B)** deletion regions of ORF3ab gene.

**Figure 3:**
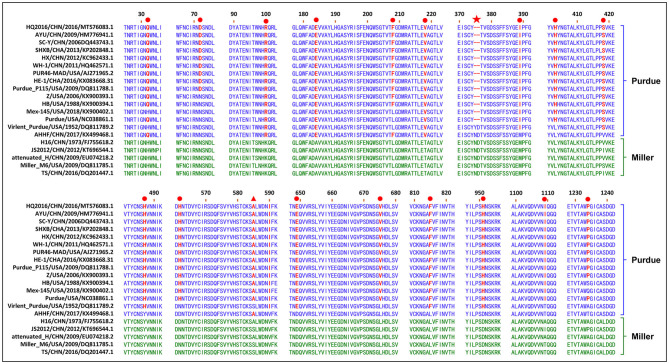
Alignment of partial deduced amino acid sequence of S protein compared with strain TGEV HQ2016. (▴) indicates amino acid 585, (★) indicates 6-nt deletion in the S gene, (•) indicates amino acids of the Purdue subgroup strains include TGEV HQ2016 are different from those of Miller subgroups strains.

The S gene of TGEV HQ2016 was 4,344-nt in length, predicted to a encode protein of 1,447 amino acids. A site of 6-nt deletion was observed in the S gene of TGEV HQ2016 at nt 1,123–1,128, which causes two amino acids shorter at this site than in strains of Virulent Purdue, AHHF, TS, Miller M6, JS2012, Attenuated H, and H16 (Figure 2A). A other site of 3-nt deletion was detected at nt 2,387–2,389 of the S gene in attenuated H, H16, and AHHF, while it was not found in strain TGEV HQ2016 and other strains (Figure 2A). In the Virulent Purdue, Miller M6, JS2012, and TS strains, amino acid 585 is serine, whereas in the TGEV HQ2016, it is alanine (Figure 3). Amino acids at 32, 72, 100, 184, 208, 218, 389, 403, 418, 487, 562, 590, 649, 675, 815, 951, 1,109, and 1,234 of TGEV HQ2016 S protein are same to those of the Purdue subgroup strains, especially the three viruses from the United States, and HE-1, HX, AYU, WH-1, SHXB, SC-Y from China, but differ from those of the Miller subgroup strains (Figure 3). The structural proteins of E, M and N were 249-nt, 789-nt and 1,149-nt in length and predicted to encode proteins of 82, 262, and 382 amino acids, respectively (Table 3), and there was no deletions or insertions compared with other TGEV reference strains.

**Table 3:** Length of amino acids in the predicted structural and non-structural proteins of TGEV strains.

**Strain**	**ORF1a**	**ORF1b**	**S**	**ORF3a**	**ORF3b**	**E**	**M**	**N**	**ORF7**
SHXB	4017	2678	1447	71	244	82	262	382	78
Purdue P115	4017	2678	1447	71	244	82	262	382	78
PUR46-MAD	4017	2678	1447	71	244	82	262	382	78
WH-1	4017	2678	1447	71	244	82	262	382	78
AYU	4017	2678	1447	71	244	82	262	382	78
Purdue	4017	2678	1447	71	244	82	262	382	78
HX	4017	2678	1447	71	244	82	262	382	78
HE-1	4017	2678	1447	71	244	82	262	382	78
SC-Y	4017	2678	1447	71	244	82	262	382	78
Z	4017	2678	1447	71	244	82	262	382	78
HB	4017	2678	1447	71	244	82	262	382	78
Mex145	4017	2678	1447	71	244	82	262	382	78
Virulent Purdue	4017	2678	1449	71	244	82	262	382	78
AHHF	4017	2678	1448	71	244	82	262	382	78
TS	4017	2678	1449	65	244	82	262	382	78
JS2012	4017	2678	1449	65	244	82	262	382	78
Miller M6	4017	2678	1449	65	244	82	262	382	78
Attenuated H	4017	2678	1448	65	244	82	262	382	78
H16	4017	2678	1448	65	244	82	262	382	78
HQ2016	4017	2678	1447	71	244	82	262	382	78

The replicase genes contained ORF1a and ORF1b, which were12,054-nt and 8,037-nt in length, predicted to encode proteins of 4,017 amino acids and a protein of 2,680 amino-acid, respectively (Table 3). There were a common 43-nt region (nt 12,315–12,357) between ORF1a and ORF1b, and a “slippery site” (5′-UUUAAAC-3′, nt 12,322–12,328) which allows the ORF1a translation termination site to be bypassed and an additional ORF, ORF1b to be read. Nucleotide sequence analysis indicated that there were no major deletions or insertions presented in replicase genes both in any Purdue and Miller TGEV strains. ORF3a and 3b of TGEV HQ2016 are216-nt and 735-nt in length, predicted to encode a protein of 71 amino acid and a protein of 244 amino acid, respectively (Table 3). Previous research had demonstrated the presence of two deletions in the TGEV ORF3a/b gene in the Miller subgroup (5), a 16-nt deletion and a 29-nt deletion were observed in the strains of Miller subgroup in this study (Figure 2B), but no deletions were detected in the ORF3a/b genes of TGEV HQ2016 and other Purdue strains. The ORF7 gene of TGEV HQ2016 was 237-nt in length and predicted to encode a protein of 78 amino acid, which contains the common PP1c-binding motif 5′-RVIFLVI-3′. No deletions or insertions presented in ORF7 of TGEV HQ2016. The recombination analysis showed that no recombination event has ever occurred in TGEV HQ2016. Complete sequence alignment of 5′ and 3′-UTR regions, there was no deletions or insertions were found in strain HQ2016. The ORF initiated by short AUG beginning within the Kozak sequence (TCTATGA) in 5′ NTR regions, and the octameric sequence of “GGAAGAGC” at upstream of the 3′ end poly(A) tail, which could be found in all strains.

### Phylogenetic Tree and Homology Analysis

**Figure 4:**
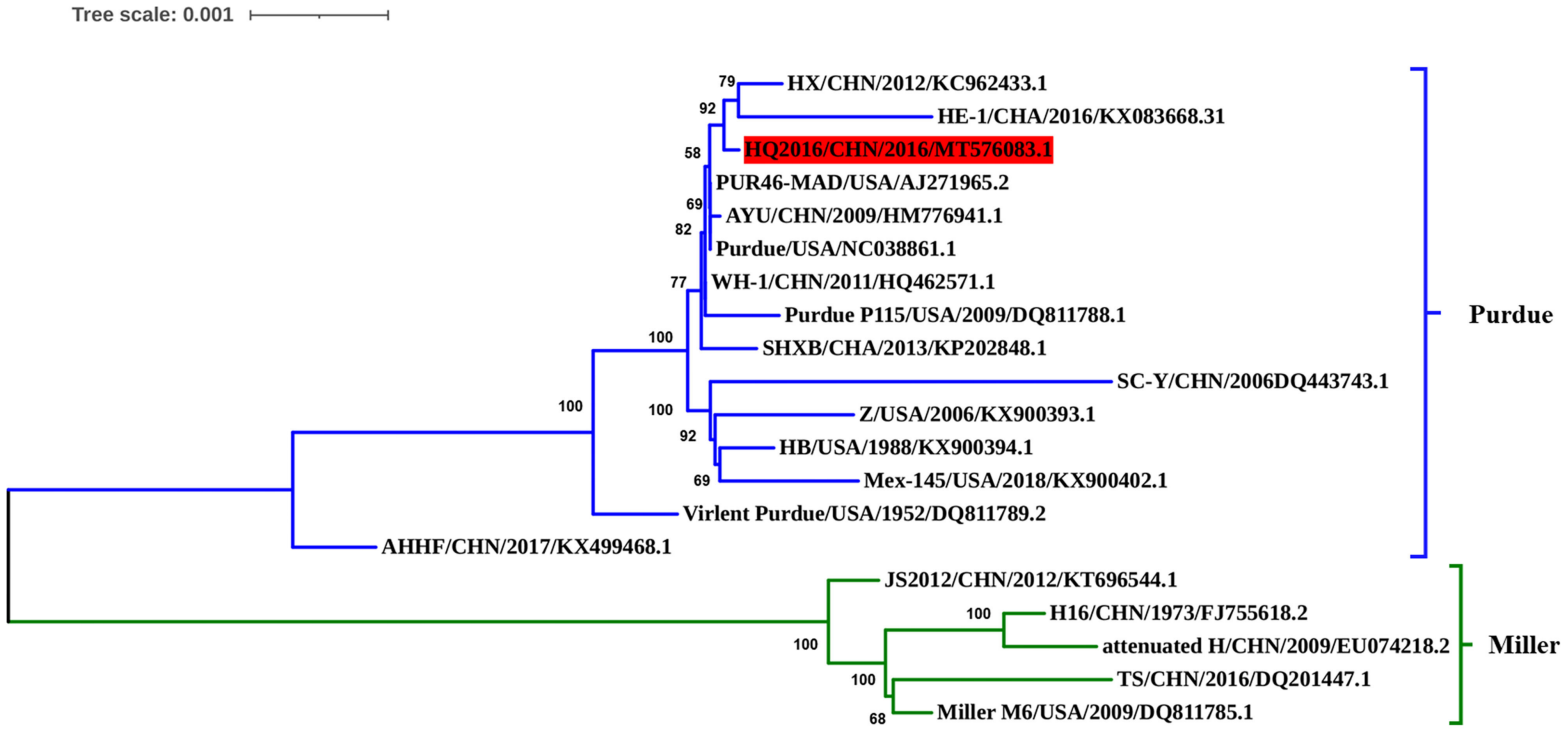
Phylogenetic analysis of the complete genome sequences of the strain HQ2016, other TGEV reference strains. TGEV HQ2016 belongs to the Purdue cluster of TGEV, not the Miller cluster. Complete genome were aligned used Clustal W program which have trimed both 3′ and 5′ ends gaps between TGEV genomes Phylogenetic tree was constructed using the neighbor-joining method with the MEGA 6.0 program. The optimal tree with the sum of branch length = 0.02540989 is shown. The percentage of replicate trees in which the associated taxa clustered together in the bootstrap test (1,000 replicates) are shown next to the branches. The tree is drawn to scale, with branch lengths in the same units as those of the evolutionary distances used to infer the phylogenetic tree. The evolutionary distances were computed using the Tajima-Nei method.

The complete genomic sequence of TGEV HQ2016 was compared with those of 19 TGEV reference strains. Phylogenetic trees based on the complete genome (Figure 4) divided the TGEV strains into the Purdue and Miller genotypes (5). The TGEV HQ2016 strain clustered in the Purdue subgroup, together with SHXB, Purdue, Purdue P115, PUR46-MAD, WH-1, AYU, SC-Y, HX, HE-1, Z, HB, Mex145, Virulent Purdue, and AHHF, whereas the Miller subgroup included TS, JS2012, Miller M6, Attenuated H, and H16. Thus, TGEV strain HQ2016 is closely related to the Purdue strains and more distantly to the Miller strains. The strains of Purdue subgroup appear to share a common ancestor.

To investigate the homology of TGEV HQ2016 with other TGEVs, the nucleotide and predicted amino acid sequences of structural proteins and non-structural proteins were compared (Table 4). The results shown that structural proteins (S, E, M, N) and non-structural proteins (replicases 1a and 1b, ORF 3a and 3b, ORF 7) of TGEV HQ2016 shared greater identity with Purdue strains (Table 4), identity of predicted amino acid sequence identity in ORF1a was 98.7–100%, in ORF1b was 98.6–100%, in S protein was 97.1–100%, in ORF3a was 88.3–100%, in ORF3b was 97.1–100%, in E protein was 91.5–98.8%, in M protein was 97.3–99.6%, in N protein was 98.2–100%, in ORF7 was 93.6–100%.

**Table 4:** Nucleotide and amino acid sequence identities (%) of TGEV HQ2016 strain compared with other 19 TGEV strains.

	**ORF1a**	**ORF1b**	**S**	**ORF3a**	**ORF3b**	**E**	**M**	**N**	**ORF7**
SHXB	99.9/99.9	100.0/100.0	100.0/100.0	100.0/100.0	99.9/99.6	99.2/97.6	99.7/99.2	99.9/99.7	99.3/97.4
Purdue P115	99.9/99.9	100.0/100.0	99.9/99.9	100.0/100.0	99.9/99.6	99.6/98.8	99.9/99.6	99.9/99.7	100.0/100.0
PUR46-MAD	100.0/100.0	100.0/100.0	100.0/100.0	100.0/100.0	100.0/100.0	99.6/98.8	99.9/99.6	100.0/100.0	100.0/100.0
WH-1	100.0/100.0	100.0/100.0	100.0/100.0	100.0/100.0	99.9/99.6	99.6/98.8	99.9/99.6	100.0/100.0	100.0/100.0
AYU	99.9/99.9	100.0/100.0	100.0/100.0	100.0/100.0	100.0/100.0	99.6/98.8	99.7/99.2	100.0/100.0	100.0/100.0
Purdue	100.0/100.0	100.0/100.0	100.0/100.0	100.0/100.0	100.0/100.0	99.6/98.8	99.9/99.6	100.0/100.0	100.0/100.0
HX	99.9/99.9	100.0/100.0	99.9/99.9	100.0/100.0	100.0/100.0	99.6/98.8	100.0/100.0	100.0/100.0	100.0/100.0
HE-1	99.9/99.7	99.8/99.7	99.9/99.8	100.0/100.0	100.0/100.0	98.8/98.8	99.5/98.5	99.9/99.7	99.8/98.7
SC-Y	99.5/99.2	99.8/99.8	99.7/99.5	100.0/100.0	99.9/99.6	99.6/98.8	99.7/99.2	99.9/99.7	100.0/100.0
Z	99.9/99.8	99.9/99.9	99.6/99.0	99.1/98.6	99.9/99.6	99.2/98.8	99.7/99.2	99.8/99.7	100.0/100.0
HB	99.9/99.9	100.0/100.0	99.7/99.4	100.0/100.0	99.9/99.6	99.6/98.8	99.7/99.2	100.0/100.0	100.0/100.0
Mex145	99.9/99.8	99.9/99.9	99.7/99.2	99.5/98.6	99.9/99.6	99.2/98.8	99.7/99.2	99.9/99.7	100.0/100.0
Virulent Purdue	99.9/99.7	100.0/100.0	99.5/99.1	99.5/98.6	99.7/99.2	99.2/97.6	99.7/99.2	99.7/99.7	100.0/100.0
AHHF	99.5/99.5	100.0/100.0	98.9/98.6	100.0/100.0	99.9/99.6	99.6/98.8	99.7/99.2	100.0/100.0	100.0/100.0
TS	98.8/98.7	99.0/98.6	98.3/98.1	87.0/89.5	98.5/96.3	98.4/95.1	98.0/96.9	98.1/98.2	96.8/93.6
JS2012	99.0/99.1	99.0/99.7	98.6/98.3	88.0/88.7	98.8/97.1	98.4/95.1	98.2/97.7	98.2/98.4	96.8/93.6
Miller M6	99.0/99.1	99.1/99.6	98.3/97.1	88.0/88.3	98.9/97.5	98.0/93.9	98.2/97.7	98.2/98.4	96.6/93.6
Attenuated H	98.9/98.9	99.0/99.6	98.0/97.7	87.5/88.7	98.8/97.1	96.8/91.5	98.1/97.3	98.1/98.4	96.8/93.6
H16	98.9/98.9	99.0/99.6	98.2/97.9	88.0/88.7	98.9/97.5	97.6/93.9	98.1/97.3	98.2/98.4	96.8/93.6

### Clinical Signs in TGEV HQ2016 Inoculated Piglets

**Figure 5:**
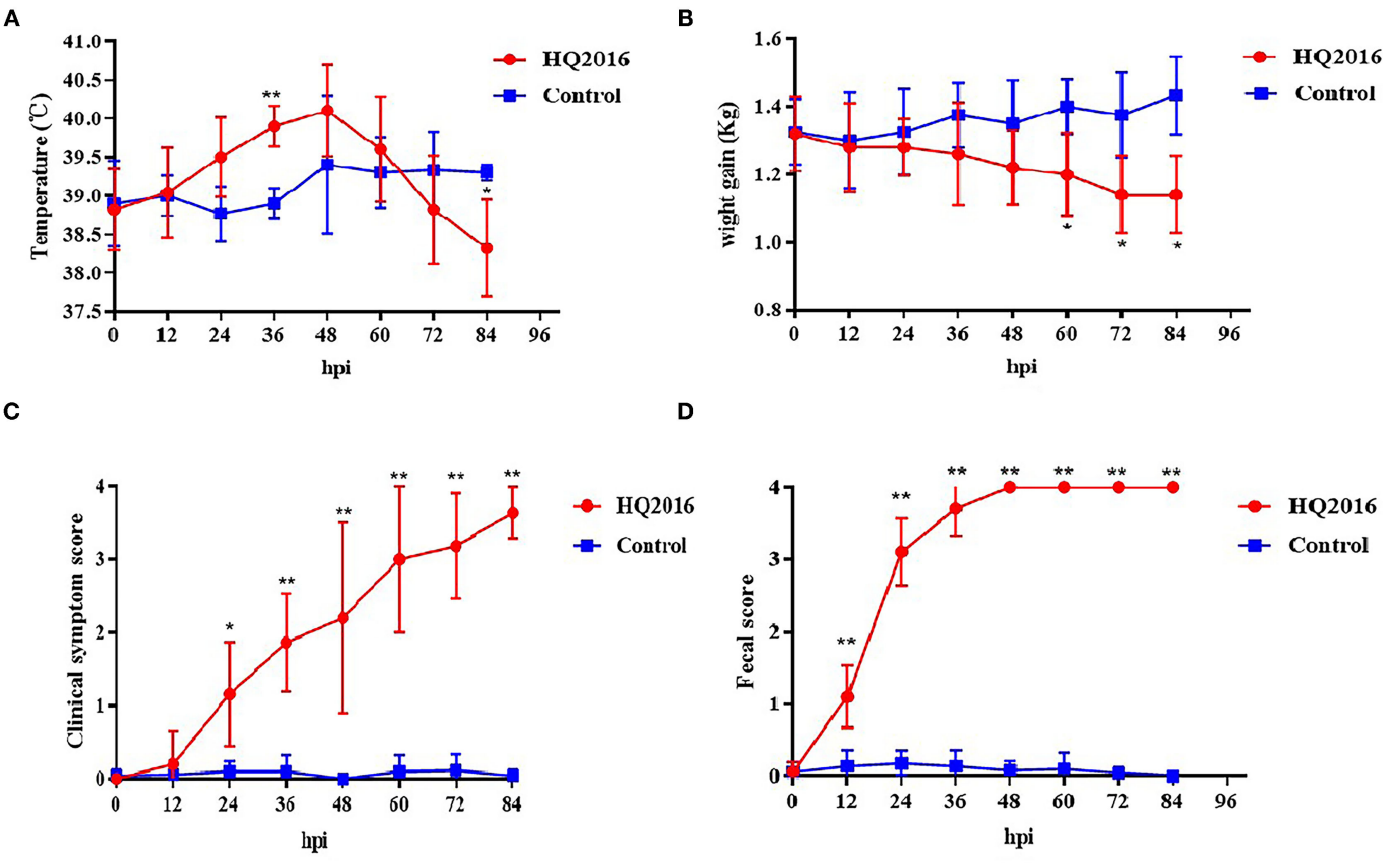
The clinical symptom in the piglets. **(A)** The temperature changes in different groups. **(B)** The body weight changes in different groups. **(C)** The clinical symptom scores in different groups. **(D)** The fecal scores in different groups. Data are shown as mean standard (*p < 0.05, **p < 0.01).

To evaluate the pathogenicity of TGEV HQ2016 in piglets, 12 newborn piglets were used without colostrum. The piglets were active and fleshy before inoculation, with normal fecal consistency. Mild diarrhea and loss of appetite were observed in the piglets of the TGEV HQ2016 inoculated group after 12 h. Severe depression, loss of appetite, vomiting, and yellow and white watery diarrhea appeared in the TGEV HQ2016 inoculated group after 48 h. After 72 h, all the piglets in TGEV HQ2016 inoculated group suffered watery diarrhea and were seriously dehydrated. None of the piglets inoculated with TGEV HQ2016 died within the 84 h of the experimental period, and the control piglets showed no vomiting or diarrhea. The body temperatures, body weight changes, clinical symptoms, and fecal scores of both groups are shown in Figure 5. The body temperatures and body weight changes were significantly lower in the piglets of the TGEV HQ2016 inoculated group after 72 h. The clinical symptoms and fecal scores increased continuously for 24 h after TGEV HQ2016 inoculated and differed significantly from those in the control group.

### Histopathological Observations

**Figure 6:**
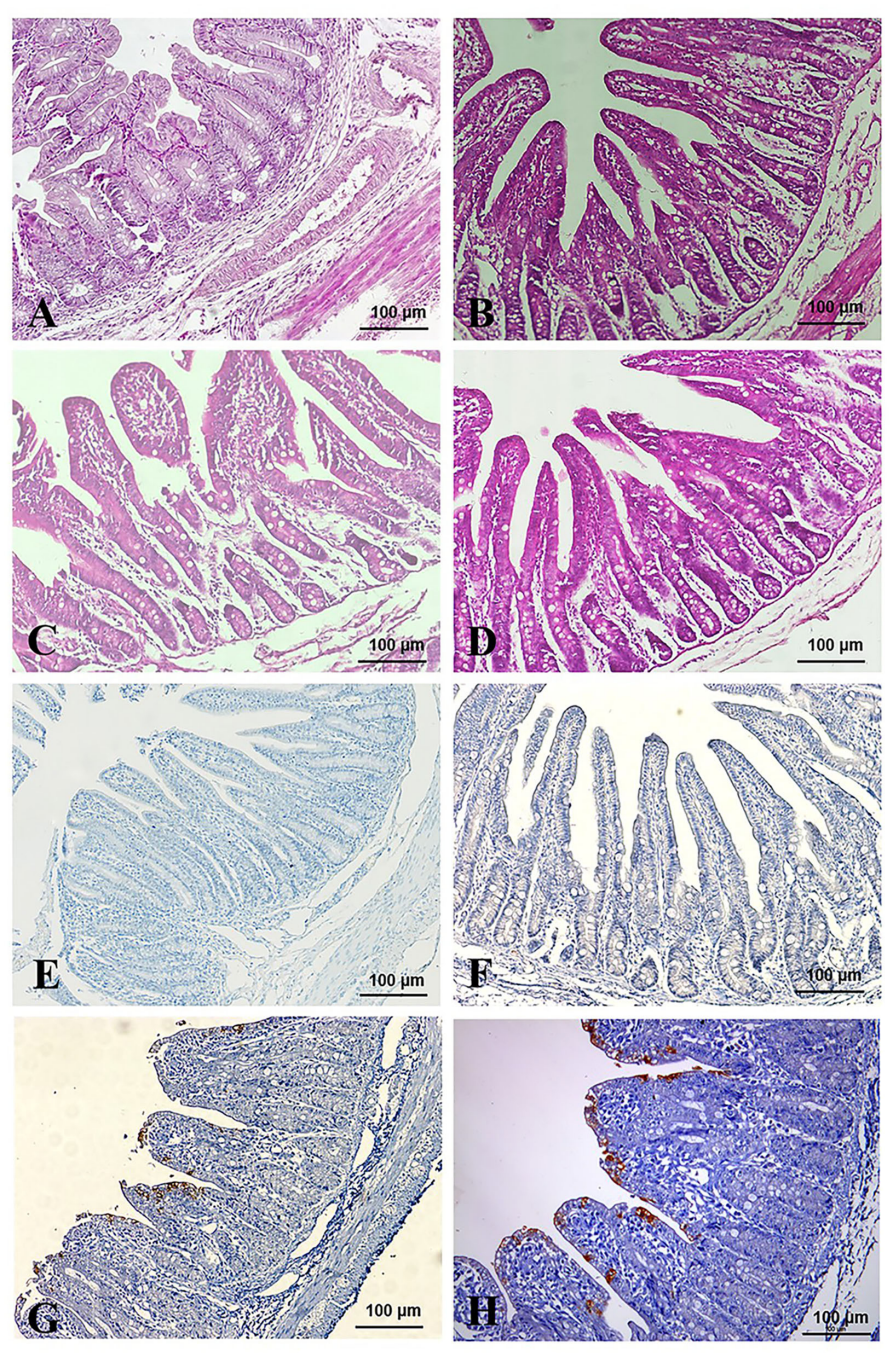
Pathological changes and IHC assays of TGEV HQ2016-inoculated piglets. **(A,B)** H.E staining for jejunum and ileum tissue section of control piglets. **(C,D)** H.E staining for jejunum and ileum tissue section of TGEV HQ2016 challenged piglets. Villus atrophy, degenerate mucosal epithelial cells, and necrosis. **(E,F)** IHC assays for jejunum and ileum tissue section of control piglets. **(G,H)** IHC assays for jejunum and ileum tissue section of TGEV HQ2016 challenged piglets. Positive cells presented in the epithelial cells in the atrophied villi of the segments of jejunal and ileal tissues from the piglets.

All the piglets were sacrificed after virus challenged 84 h. Pathological changes were mainly observed in the intestinal tracts (jejunum and ileum) of the TGEV-HQ2016-challenged piglets. The whole intestinal tracts, in which yellow watery contents had accumulated, were transparent, thin walled, and gas distended. No lesions were observed in any other organs of the TGEV HQ2016 inoculated piglets or in the organs in the negative control piglets, indicating that the intestinal tract is the target organ of TGEV infection. In a microscopic examination, villus atrophy, degenerate mucosal epithelial cells, and necrosis were observed in both the jejunum and ileum tissues of the TGEV HQ2016 inoculated piglets, but not in those of the control piglets, as shown in Figure 6. An immunohistochemical examination showed TGEV antigen in the cytoplasm of the epithelial cells in the atrophied villi of the segments of jejunum and ileum tissues from the piglets inoculated with TGEV HQ2016, but no reactivity in either the jejunal or ileal tissues of the control group, as shown in Figure 6.

### Viral Loads in Fecal Samples and Intestinal Tissues of TGEV HQ2016 Inoculated Piglets

**Figure 7:**
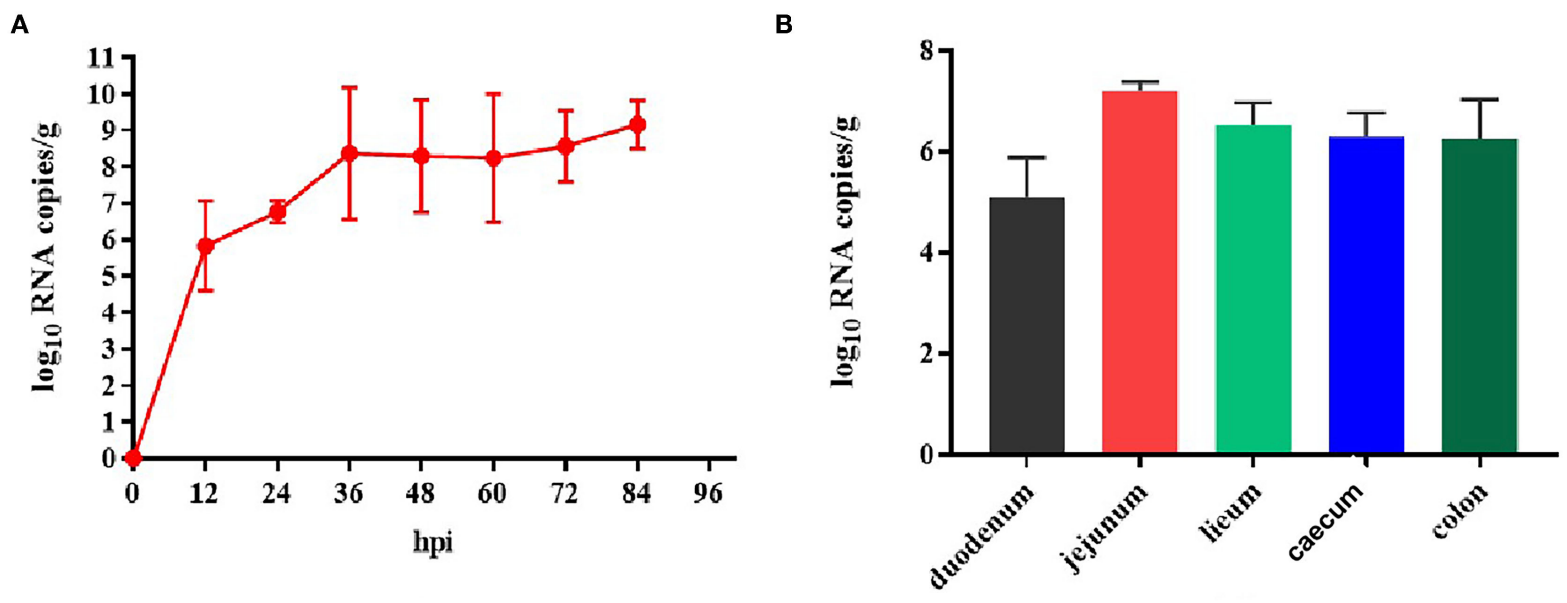
Reproduction of watery diarrhea and viral shedding in newborn piglets inoculated with TGEV HQ2016 via oral feeding. **(A)** Quantification of viral RNA levels of fecal samples of piglets inoculated with TGEV HQ2016. **(B)** Quantification of viral RNA levels in intestine tissues of piglets at 84 h inoculated with TGEV HQ2016.

Because TGEV caused diarrhea and intestinal damage in the newborn piglets, we collected rectal swabs and intestinal samples from them to investigate the viral shedding in the TGEV HQ2016 inoculated piglets. White and yellow watery feces were present in the TGEV HQ2016 inoculated piglets from 48 h after virus challenged. As shown in Figure 7, the TGEV viral RNA was detected with quantitative RT-PCR (19). The TGEV levels in the fecal samples were 5–10 log_10_ RNA copies/g at 12–84 hpi, indicating that TGEV HQ2016 infected and reproduced in these challenged piglets. At the end of the challenge experiment, samples of duodenum, jejunum, ileum, caecum, and colon were collected for viral RNA detection. At 84 hpi, the viral level was highest in the jejunum (7.21 ± 0.11 log_10_ RNA copies/g), and then (in decreasing order) in the ileum (6.51 ± 0.31 log_10_ RNA copies/g), cecum (6.28 ± 0.39 log_10_ RNA copies/g), colon (6.23 ± 0.55 log_10_ RNA copies/g), and duodenum (5.09 ± 0.61 log_10_ RNA copies/g). These results confirm that TGEV HQ2016 infected the piglets and invaded their intestinal tissues.

## Discussion

TGEV is an enteropathic coronavirus that infects pigs, and was first reported in the USA in the 1940s, after which spread throughout the world (1–3). TGEV causes significant diarrhea, vomiting, and dehydration in suckling piglets, with a high mortality rate (10). In recent years, mixed infections of TGEV with other swine diarrhea virus have occurred frequently, causing serious economic losses in the pig industry (1). In this study, a natural strain of TGEV, HQ2016, was successfully isolated from piglets intestinal samples, which collected from swine-raising farms in northeast China. In the farms, sows did not receive any vaccination for preventing diarrhea and piglets developed clinical symptoms including vomiting, diarrhea, rapid weight loss and dehydration. After experimental infection, piglets showed the characteristic clinical symptoms (diarrhea and vomiting) of TGE from 12 h after TGEV HQ2016 inoculated until the end of the experiment. A histopathological analysis showed villous atrophy, together with mucosal epithelial cells degeneration and necrosis, in the jejunum and ileum, and virus-positive cells were present in the villous epithelial cells in the jejunum and ileum by IHC. These results demonstrate that TGEV HQ2016 was replicated and had pathogenicity in enterocyte, is a natural, transmissible, enteric pathogenic porcine coronavirus. Viral nucleic acid of TGEV was detected on rectal swabs as early as 12 h after viral challenge, which indicated that virus infected the intestine and released to intestinal content, as described previously in infections with TGEV (6, 20). At 84 h of TGEV HQ2016 inoculated, we found a high level of viral RNA in jejunum, ileum, caecum and colon, which is similarity with the report previously (5), but there was no obviously pathological changes and TGEV antigen presence in caecum and colon epithelial cells (which is not shown in the results of this study), this result suggested that caecum and colon contained virus but epithelial cells had not yet been infected. Virus-positive epithelial cells and presence of virus in intestines indicated that TGEV HQ2016 prefers to infect small intestinal epithelial cells and replicate, caused pathological changes in the small intestinal epithelial cells, and then necrotic epithelial cells released the virus into the intestinal contents, and finally excreted through the large intestines. This finding may provide a proof for the study of host cell infection and transmission mechanism in coronavirus.

Traditional TGEVs can be divided into two clusters, the Purdue and Miller groups (4, 5, 7, 12, 21). In this study, we sequenced the entire genome of TGEV HQ2016, and a phylogenetic analysis placed TGEV HQ2016 in the Purdue cluster, indicating that it is more distantly evolutionarily related to the Miller cluster. Additionally, sequence alignment result showed two large deletions in ORF3a/3b that occur in the strains of the Miller cluster are not found in TGEV HQ2016 or the Purdue cluster, this may be considered to a marker of distinguishing the Purdue and Miller cluster of TGEV. Phylogenetic analysis shown that TGEV HQ2016 is closely related to with strains PUR46-MAD, Purdue, WH-1, AYU, which have the same ancestor, and this is consistent with the results of homology comparison. Nucleotide and predicted amino-acid sequence homology comparison shown the structural and non-structural proteins of TGEV HQ2016 is very similar to PUR46-MAD, Purdue, AYU and WH-1. These data suggest that TGEV HQ2016 might be had the same origin with WH-1 and AYU strains in China and more similar with Purdue and PUR46-MAD from USA.

The 5′- and 3′-UTRs of CoVs are critically important for viral replication and transcription (5, 22, 23). The “slippery” heptanucleotide sequence and a pseudoknot structure are both critical for viral RNA synthesis and are involved in ribosomal frame shifting (24). A complete sequence analysis indicated that no deletions or insertions are present in the 5′- or 3′-UTR regions of TGEV HQ2016, and that it contains both the slippery sequence and pseudoknot structure. These sequence data suggest that the replication and transcription mechanisms of TGEV HQ2016 are conserved, as reported previously (5, 21, 25).

CoVs attach to their host cells via the S protein, which is the major immunogenic protein of the virus and stimulate the host to produce antibodies with neutralizing activity (26). There are at least four main antigenic sites on the S protein, designated A, B, C, and D (4, 27, 28). The A/B sites (amino acids 506–706) are the major antigenic sites and have been mapped. Single-amino-acid changes in the S protein might affect its antigenicity or virulence (4–6). A mutation at amino acid 585 in the main major antigenic sites A/B of the S protein of TGEV HQ2016 causes a serine to alanine change, which also occurs in the PUR46-MAD, Purdue, Purdue P115, WH-1, AYU, HX, HE-1, SHXB, SC-Y, Z, HB, Mex145, AHHF, H16, and Attenuated H strains, but not in the JS2012, Miller M6, TS, or Virulent Purdue strains. This mutation may significantly influence receptor binding or the virus interactions with neutralizing antibodies, significantly affecting their antigenicity, this is also considered to be a marker of attenuation (6). There was a 6-nt deletion detected in the TGEV HQ2016 S gene, as in the rest of the Purdue cluster, except for the Virulent Purdue and AHHF strains. A 6-nt deletion (nt 1,123-1,128) in the S gene was considered a trait of the TGEV strains in the Purdue cluster (5). This 6-nt deletion in the S gene was also considered to play a role in viral attenuation (6). The S gene is also a hypervariable region in the TGEV genome, and amino acids 32, 72, 100, 184, 208, 218, 389, 403, 418, 487, 562, 590, 649, 675, 815, 951, 1,109, and 1,234 of TGEV HQ2016 are identical among the viruses in the Purdue cluster, but differ from those in the Miller cluster. These changes of amino acid in S gene may be related to the changes of virus virulence, which needs to be discussed in follow-up research. Except for S gene, ORF3a/3b genes were considered to affect the variation between attenuated and virulent strains (12). However, there are some uncertainties about the effects of deletions in TGEV ORF3a/3b on viral virulence (1, 28–31). In our study, homology analysis shown that HQ2016 and attenuated strains PUR46-MAD (4, 32) had highly identity. PUR46-MAD was generally considered an attenuated strain of TGEV, which derivative of Purdue P115, and both were derived from the strain virulent Purdue after highly passage in cell culture (4, 12, 25, 32, 33). TGEV HQ2016 used in our infected experiment was only 10th passage in cell culture. Therefore, we think that the virulence of HQ2016 might be reduced by highly passage in cell culture in the future, as previously reported for PUR46-MAD. 6-nt deletion or amino acid mutations in S gene might reduce the virulence of TGEV HQ2016 through the highly passage, this need to be confirmed in future studies. This hypothesis needs to be confirmed in future studies and facilitate the development of an attenuated vaccine for TGEV.

In conclusion, a epidemical strain of TGEV, HQ2016, was isolated from swine-raising farms in northeast China. Typical clinical signs, pathologic alterations and histological changes associated with TGE were observed in piglets inoculated with the TGEV HQ2016 strain. Phylogenetic analysis of whole genome, nucleotide and amino acid sequence homology analysis of the structural proteins and non-structural proteins indicated that TGEV HQ2016 belongs to the Purdue cluster, and it might be had the same origin with WH-1 and AYU strain in China and more similar with Purdue strains from USA. These results provide essential information for further understanding the evolution of TGEV and will facilitate future investigations into the molecular pathogenesis of TGEV.

## Data Availability Statement

The datasets generated in this study can be found in online repositories. The names of the repository/repositories and accession number(s) can be found below: https://www.ncbi.nlm.nih.gov/genbank/, MT576083.

## Ethics Statement

The animal study was reviewed and approved by Animal Experiment Ethical Committee of Heilongjiang Bayi Agricultural University.

## Author Contributions

DY: formal analysis and writing—original draft. ZY: methodology and validation. ML: methodology. YW: data curation. MS: writing and picture editing. DS: supervision. All authors contributed to the article and approved the submitted version.

## Conflict of Interest

The authors declare that the research was conducted in the absence of any commercial or financial relationships that could be construed as a potential conflict of interest.
